# Case report: A case of malignant phyllodes tumor of the breast with concurrent epithelial malignancy and heterologous chondrosarcomatous differentiation

**DOI:** 10.3389/fonc.2026.1807860

**Published:** 2026-04-24

**Authors:** Jie Yuan, Hua Zhang, Jun Huang, Bei Wang, Geng Wang, Li Yang

**Affiliations:** 1 Department of Breast, Thyroid and Vascular Surgery, Hubei Provincial Clinical Research Center for Umbilical Cord Blood Hematopoietic Stem Cells, Taihe Hospital, Hubei University of Medicine, Shiyan, Hubei, China; 2 Department of Clinical Laboratory Medicine, Taihe Hospital, Hubei University of Medicine, Shiyan, Hubei, China

**Keywords:** breast neoplasms, chondrosarcoma, ductal carcinoma in situ, malignant phyllodes tumor, metaplastic differentiation

## Abstract

The presence of malignant heterologous elements and malignant transformation of the epithelial component in phyllodes tumor (PT) is infrequent. The co-existence of both features within a single malignant PT is exceptionally rare and poorly documented. We report a unique case of a 57-year-old female with a malignant PT exhibiting both low-grade ductal carcinoma *in situ* (DCIS) and heterologous chondrosarcomatous differentiation. This case underscores the diagnostic challenges and therapeutic considerations for this complex tumor entity, highlighting the critical importance of extensive histopathological sampling.

## 1 Introduction

Phyllodes tumors (PTs) are uncommon fibroepithelial neoplasms, representing 0.3-0.9% of all breast tumors (1). They are characterized by a dual proliferation of epithelial and stromal components (2). The World Health Organization (WHO) classifies PTs into benign, borderline, and malignant categories based on histological features including stromal cellularity, atypia, mitotic activity, overgrowth, and margin status (3). Malignant PTs constitute 10-20% of cases and are defined by pronounced stromal atypia, high mitotic counts (≥10/10 HPF), stromal overgrowth, infiltrative borders, and/or the presence of malignant heterologous elements (4). The occurrence of malignant heterologous differentiation, such as osteosarcoma or chondrosarcoma, is rare (5). Similarly, carcinoma arising from the epithelial component of a PT is an infrequent event (6). Herein, we present a detailed clinicopathological report of a case exhibiting both types of characteristics and discuss the diagnostic, therapeutic, and prognostic implications.

## 2 Case presentation

**Figure 1:**
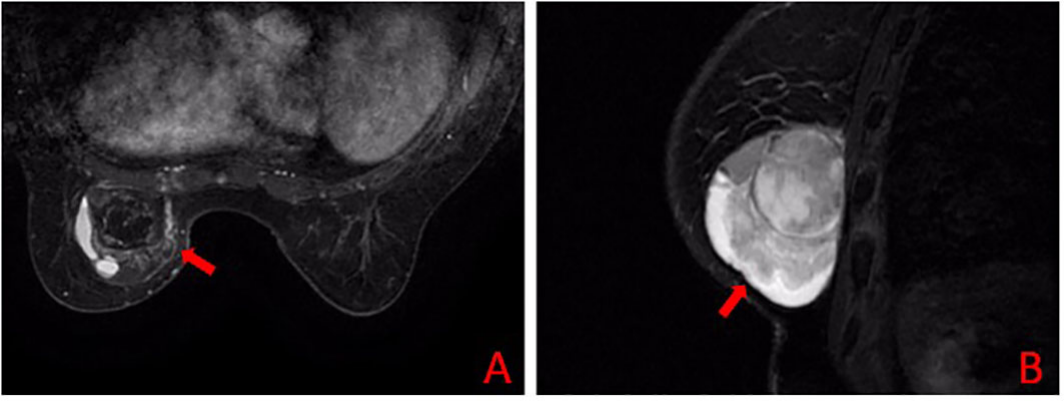
Preoperative breast MRI. **(A)** axial and **(B)** sagittal contrast-enhanced T1-weighted images show a large, lobulated, heterogeneously enhancing mass (arrows) in the left breast.

A 57-year-old female presented with a one-month history of a palpable left breast mass. Physical examination revealed a firm, approximately 6.0 × 6.0 cm mass in the 6 to 11 o’clock region, with surface protrusion and moderately defined margins. No axillary lymphadenopathy was detected. Breast ultrasonography identified a heterogeneous, hypoechoic mass measuring 61 × 61 × 45 mm, classified as BI-RADS IV. Subsequent contrast-enhanced magnetic resonance imaging (MRI) demonstrated a lobulated mass (61 × 50 × 47 mm) with mixed T1/T2 signals, diffusion restriction, and heterogeneous enhancement, leading to a BI-RADS V assessment (**Figure 1**).

An ultrasound-guided core needle biopsy was performed. Initial pathological evaluation suggested a fibroepithelial tumor with cartilaginous tissue, prompting a recommendation for complete excision. The patient underwent lumpectomy. Gross examination of the specimen revealed a well-circumscribed, 5.5 cm nodule with a variegated, grayish-white to grayish-red cut surface and focal cystic degeneration.

**Figure 2:**
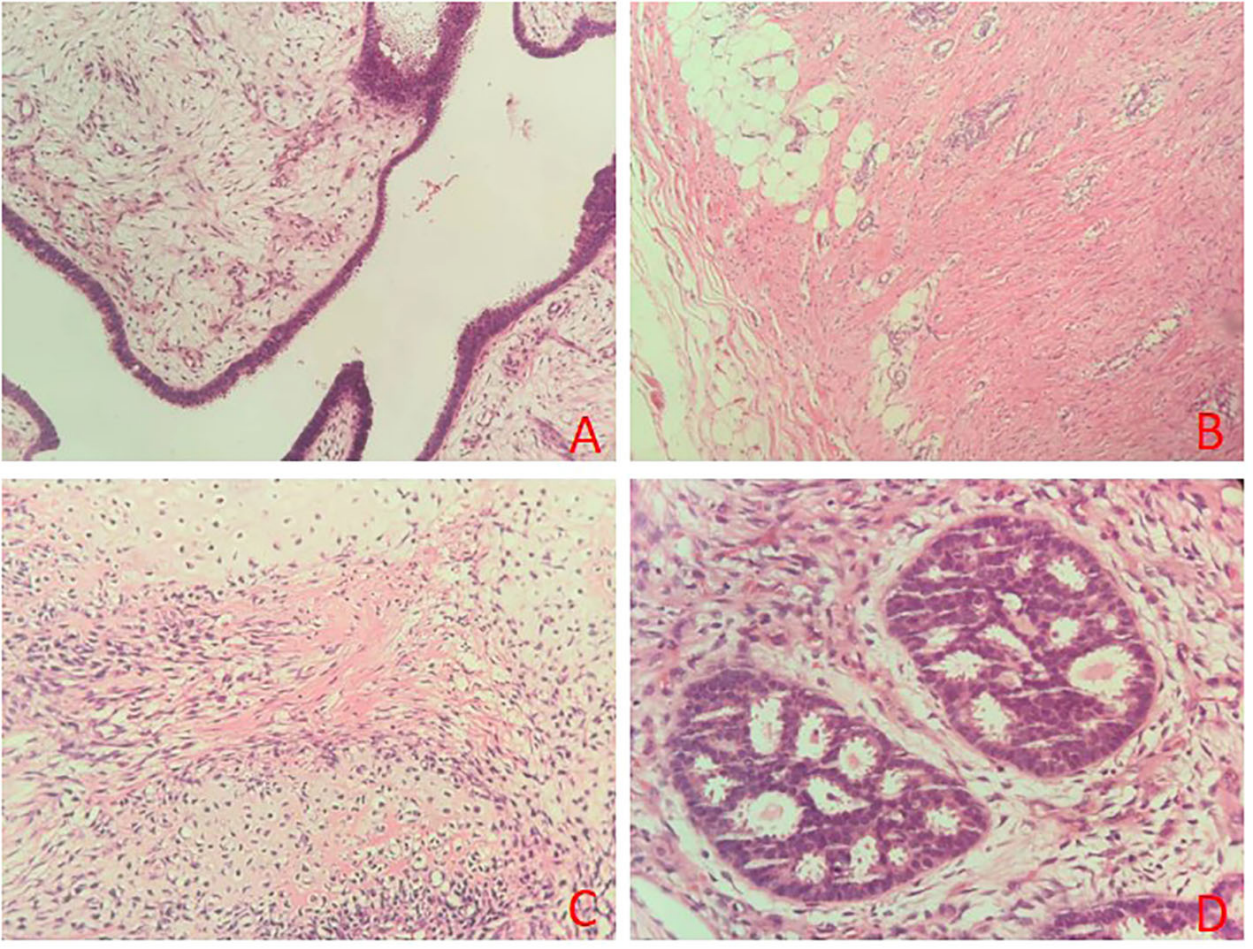
Histopathological features (hematoxylin and eosin stain). **(A)** characteristic phyllodes architecture with benign epithelial lining and cellular stroma (×100). **(B)** area of stromal overgrowth and an infiltrative tumor border (×200). **(C)** focus of well-differentiated chondrosarcoma with lacunae containing atypical chondrocytes within the stromal component (×200). **(D)** focus of low-grade ductal carcinoma *in situ* (DCIS) with a cribriform pattern (×200).

Histopathological examination confirmed the diagnosis of a malignant phyllodes tumor. The tumor displayed characteristic leaf-like architecture lined by benign, bilayered epithelium (**Figure 2A**). Areas of stromal overgrowth, a mitotic count of 12 per 10 high-power fields (HPF), and focally infiltrative tumor borders were evident (**Figure 2B**). Notably, distinct foci of heterologous, well-differentiated chondrosarcoma were identified within the stroma (**Figure 2C**). Extensive sampling revealed additional foci of low-grade ductal carcinoma *in situ* (DCIS) (**Figure 2D**). The final integrated diagnosis was malignant phyllodes tumor with chondrosarcomatous differentiation and concurrent low-grade DCIS.

Immunohistochemical analysis of the DCIS component (slide #3) showed strong positivity for estrogen receptor (ER >90%) and progesterone receptor (PR ~80%), with HER-2 score of 1+, a low Ki-67 index (~2%), and negative CK5/6. Analysis of the chondrosarcomatous area (slide #14) showed negativity for epithelial markers (CKP, P40), S100, and focal positivity for SATB2, with a higher proliferative index (Ki-67 hotspot ~20%).

Following a multidisciplinary tumor board review and detailed patient consultation, a completion left total mastectomy with sentinel lymph node biopsy was performed. The sentinel lymph node was negative for metastasis (0/1). Final pathology confirmed negative resection margins and no residual tumor. The patient received adjuvant chemotherapy with an epirubicin and ifosfamide-based regimen, which was discontinued after three cycles due to severe adverse effects (myelosuppression and gastrointestinal toxicity). Recommendations for adjuvant radiotherapy and endocrine therapy (tamoxifen) were provided based on the tumor size and hormone receptor status of the DCIS component. The patient has been undergoing follow-up examinations every six months since the surgery. The most recent follow-up, conducted approximately three years post-surgery, showed no signs of local recurrence, and no metastases were detected in distant organs (lungs, liver, and brain).

## 3 Discussion

This report describes a case of malignant PT harboring both carcinomatous (DCIS) and sarcomatous (chondrosarcoma) differentiation. The pathogenesis of heterologous elements in PTs remains debated. The prevailing hypothesis supports a metaplastic origin, whereby multipotent stromal cells undergo aberrant differentiation into osseous or chondroid lineages, with subsequent malignant transformation (7). This theory is favored over the concept of embryonic rest origin. The finding of SATB2 positivity in the chondrosarcomatous component in our case supports osteochondral lineage differentiation (8).

The malignant transformation of the epithelial component in PTs is a well-documented but rare phenomenon, most frequently manifesting as DCIS or invasive ductal carcinoma (9). The presence of low-grade, hormone receptor-positive DCIS within a malignant PT, as seen in our patient, creates a unique therapeutic dilemma. While the primary driver of aggressive behavior in malignant PT is the sarcomatous stroma, the coexistent carcinoma component necessitates consideration of adjuvant systemic therapies targeting both cellular lineages (10).

The principal differential diagnoses include mammary carcinosarcoma (metaplastic carcinoma), primary pure chondrosarcoma of the breast, and carcinoma arising within a fibroadenoma (11). Distinction from carcinosarcoma is critical. Carcinosarcoma is a monophasic, high-grade metaplastic carcinoma where both malignant epithelial and mesenchymal elements are clonally related and typically high-grade (12). In contrast, our case demonstrated a classic biphasic PT architecture with a low-grade DCIS component and a distinct heterologous chondrosarcoma arising within the stroma, supporting its classification as a malignant PT with divergent differentiation. Distinction from pure sarcoma or fibroadenoma relies on identifying the characteristic phyllodes architecture and the biphasic nature.

Management of malignant PT is primarily surgical, aiming for wide local excision with clear margins (13). The role of adjuvant therapy is not well-defined due to the rarity of the disease and lack of prospective trials (14). For conventional malignant PTs without heterologous elements or carcinoma, adjuvant chemotherapy or radiotherapy is not routinely recommended. However, the presence of a high-grade heterologous sarcoma component (e.g., osteosarcoma) or a concurrent invasive carcinoma may justify consideration of adjuvant treatment based on guidelines for soft tissue sarcomas or breast carcinoma, respectively (15). In our case, the chemotherapy regimen was chosen based on sarcoma protocols due to the chondrosarcomatous element, while endocrine therapy was indicated for the ER/PR-positive DCIS. The benefit of radiotherapy for large tumor size or close margins, though debatable, was considered (16, 17).

The prognostic significance of heterologous elements in PT is variable. Osteosarcomatous differentiation is consistently associated with a poorer outcome, while the impact of chondrosarcomatous or other heterologous types is less clear and requires further study (18). The prognosis of PTs with epithelial malignancy likely depends on the stage and grade of the carcinoma component (19). The patient in this case has been followed up for three years and has not yet shown any signs of disease progression. Long-term follow-up is essential for such rare tumors to better understand their natural history.

## 4 Conclusion

We present a highly unusual case of malignant phyllodes tumor demonstrating concurrent low-grade DCIS and heterologous chondrosarcomatous differentiation. This case highlights several key points: 1) The critical importance of extensive grossing and histological sampling in large PTs to identify rare divergent differentiations; 2) The diagnostic challenge in distinguishing such tumors from carcinosarcoma, with careful attention to architecture and grading being paramount; 3) The therapeutic complexity introduced by the dual malignant components, necessitating a multidisciplinary, tailored approach that may combine sarcoma and carcinoma management principles. This report contributes to the sparse literature on this entity and underscores the need for collaborative research to establish optimal management strategies.

## Data Availability

The original contributions presented in the study are included in the article/supplementary material, Further inquiries can be directed to the corresponding author.
